# Comparison of Measurement Models for 3D Magnetic Localization and Tracking

**DOI:** 10.3390/s17112527

**Published:** 2017-11-03

**Authors:** Guido De Angelis, Alessio De Angelis, Antonio Moschitta, Paolo Carbone

**Affiliations:** 1 Regione Umbria, 06124 Perugia, Italy; 2 Engineering Department, Università degli Studi di Perugia, 06125 Perugia, Italy; alessio.deangelis@unipg.it (A.D.A.); antonio.moschitta@unipg.it (A.M.); paolo.carbone@unipg.it (P.C.)

**Keywords:** magnetic positioning systems, magnetic measurement models, tracking, unscented Kalman filter

## Abstract

In this paper, we consider magnetic positioning and tracking of objects and provide a comparison of the characteristics of two major measurement models: the magnetic dipole model and the mutual inductance model. The numerical results obtained by applying these models to a short-range position measurement application, with a maximum operating distance of approximately 50 cm, are compared. Based on the results of this comparison, a prototype 9-sensor array is developed, experimental tests are performed, and extensive measurement results are presented. Outcomes show the feasibility of tracking the position and orientation of a mobile coil in real time with a median positioning error below 1 cm and a worst-case error of about 2 cm and 11 degrees inside a spatial region of 30 × 30 × 30 cm^3^ operational volume.

## 1. Introduction

Short-range positioning and tracking techniques are gaining increasing interest in many fields related to the Internet of Things (IoT) framework, such as indoor localization, biometrics, robotics, domotics, and health care. Applications in these fields may require high accuracy in small confined spaces. Various techniques have been proposed to solve this problem. Several approaches are based on radio propagation, such as Ultra Wide Band [1], Bluetooth [2], and Zig-Bee [3] technologies. Alternative techniques are based on the propagation of acoustic waves, such as the ultrasound techniques proposed in [4,5]. Another category of positioning technologies is represented by Magnetic Positioning Systems [6].

During the last two decades, magnetic positioning systems have attracted the attention of numerous researchers, mainly due to their robustness to propagation phenomena, such as multipath and obstructions, and due to their high potential accuracy [6]. As an example, the processing and system-level aspects of designing a magnetic positioning system based on coils and a magnetic sensor are investigated in [7]. The 3D positioning system in [8] was realized using three-axis field generating coils and a three-axis sensor coil. In [9], a method for measuring the location and orientation of a sensor based on a rotating magnetic dipole was proposed. Moreover, in [10] the authors propose a system based on three-axis magnetic coils, where each coil has outer size 5 cm × 5 cm × 5 cm and 300 turns. In [11], three transmitter coils are placed in an orthogonal arrangement with a pyramidal shape with base diameter of 21 cm and height of 11 cm. In [12] the authors use a cylindrical magnet of diameter 1/8 inches and 3/8-inch length.

In general, one of the fundamental issues is that of developing a system with reduced dimensions of the transmitters and sensors, while at the same time achieving the high positioning accuracy granted by the magnetic technology. With this aim, a system for tracking a 0.9-mm diameter subminiature coil is presented in [13]. This system is composed of a 2D-array of 64 transmitting coils, each having a diameter of 2.6 cm.

In this paper, we address the magnetic positioning and tracking problem from a measurement modeling point of view. Specifically, we consider the tracking of the pose, i.e., position and orientation, of a mobile field-generating coil by processing measurements of the voltage induced in an array of known-pose sensing coils.

The goal of the research activity presented in this paper is to analyze the implications of two different modeling techniques on positioning accuracy and computational complexity. These are the *magnetic dipole model* and the *mutual inductance model*. Either one of them forms the basic building blocks necessary to position and track systems. Thus, the analysis described in this paper can assist a system developer in making informed design choices.

In the literature, the magnetic dipole model is widely used for realizing positioning systems. However, this model may provide inaccurate results for some geometrical configurations, such as when the distance between the center of the field generator coil and that of the sensing coil is of the same order of magnitude as the diameter of the larger of the two coils, or smaller. Thus, the study of alternative models that offer better accuracy is relevant. Such models are based on the computation of the mutual inductance between coils. This comparison between the magnetic dipole model and the mutual inductance model in terms of positioning accuracy and computation time has not been provided in the literature. Therefore, it represents a novel contribution of this paper where, at first, the numerical results obtained by applying these two models are compared. Then, based on this comparison, a prototype system is realized, experimental tests are described and extensive measurement results are presented, to prove the feasibility of a practical short-range tracking system. In the context of this paper, a tracking system is considered short-range if it has a maximum operating distance of approximately 50 cm. This class of systems may be employed in such applications as hand-tracking for robotic telemanipulation, or in the biomedical field [14,15]. For these applications, compared to optical or ultrasound systems, magnetic-field positioning systems offer the advantage of being robust to obstructions and non-line-of-sight situations, e.g., due to the position of the hand that might block the view of the fingers.

The paper is structured as follows. In Section 2, the measurement models for magnetic positioning systems are presented. In Section 3, numerical results are analyzed. In Section 4, the realized magnetic tracking system is described. Finally, in Section 5, experimental results are described.

## 2. Measurement Models for Magnetic Positioning Systems

We consider a positioning system comprised of several sensing coils, having fixed and known positions, and a mobile field-generating coil. The goal is to estimate the position and orientation of the field-generating coil, based on the measured voltage at the sensing coils. In order to define the measurement model, let us consider a field-generating coil and a sensing coil with arbitrary relative position and orientation in space. The magnetic field generated by a coil driven by an alternate current induces a voltage on the sensing coil. The root-mean square value of the induced voltage may be measured at the sensing coil. Then, by collecting simultaneous measurements from multiple known-position sensing coils, it is possible to estimate the position and orientation of the field-generating coil relative to the coordinate frame of the sensing coils. The equations that relate the measured voltage at the sensing coil to the relative position and orientation of the field-generating coil with respect to the sensing coil may be derived using the model employed in [8,13], based on the concept of magnetic dipole moment. Alternatively, they may be derived by using the model in [16], based on the mutual inductance concept, or that presented in [17,18], which is an extension of the mutual inductance model in [16]. In the following subsections, the derivations of these measurement models are provided.

### 2.1. Magnetic Dipole Model

**Figure 1:**
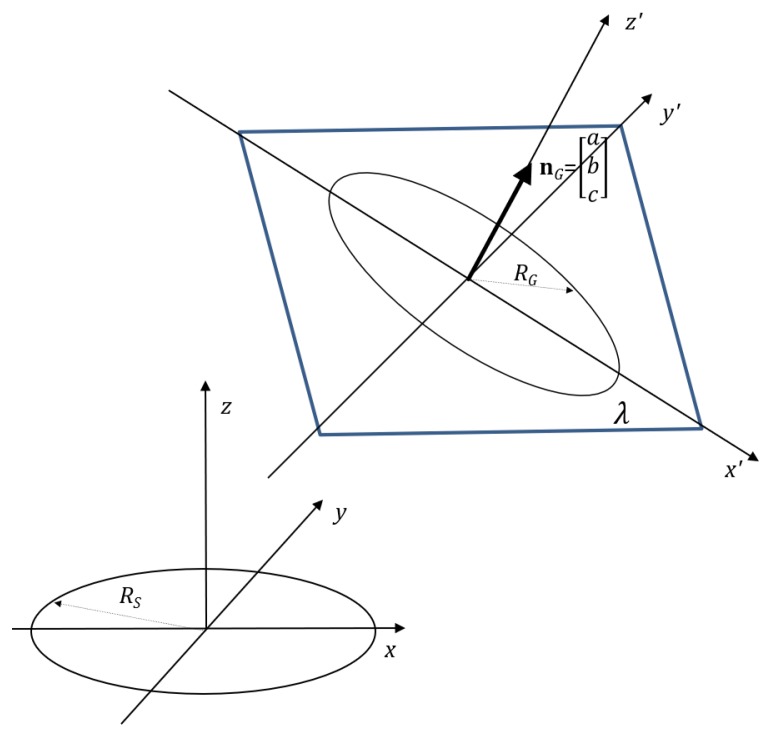
3D geometric configuration of the circular filaments in the mutual inductance model.

Assume that the sensing coil is centered at the origin of the coordinate system (see Figure 1). The field-generating coil may be approximated as a magnetic dipole. When the distance between the field-generating coil and the sensing coil is small compared to wavelength (quasi-static approximation), the magnetic flux density vector at the sensing coil, denoted as B, is given by [8,13]:

(1) $$B\left(x_{G},y_{G},z_{G},a,b,c\right)=\frac{\mu_{0}}{4\pi }\left(\frac{3\left(m\cdot r\right)r}{r^{5}}-\frac{m}{r^{3}}\right)e^{-j\omega_{0}t},$$

where $\mu_{0}$ is the magnetic permeability of vacuum, m is the magnetic dipole moment vector of the field-generating coil, $r=[x_{G},\;y_{G},\;z_{G}]$ is the vector from the center of the field-generating coil to the center of the sensing coil, r, with |r|=r, $\omega_{0}$ is the angular frequency of the sinusoidal excitation of the field-generating coil, and *t* is time. The magnetic dipole moment vector m of the field-generating coil depends on the construction of the coil, on the amplitude of the feeding current, and on the orientation of the coil. It is given by:

(2) $$m=N_{G}S_{G}In_{G}$$

where $N_{G}$ is the number of windings of the field-generating coil, $S_{G}$ is its area, *I* is the amplitude of the feeding current, and $n_{G}$ is the unit vector normal to the plane where the field-generating coil lies (thus aligned with the coil’s axis), with $n_{G}={\left[a,\;b,\;c\right]}^{T}$, as illustrated in Figure 1.

By assuming a uniform flux inside the sensing coil, the root-mean-square (rms) voltage at the sensing coil, denoted as $V_{S}$, may be obtained as [19]

(3) $$V_{S}\left(x_{G},y_{G},z_{G},a,b,c\right)=\omega_{0}N_{S}S_{S}B\left(x_{G},y_{G},z_{G},a,b,c\right)\cdot n_{S}$$

where $N_{S}$ is the number of windings of the sensing coil, $n_{S}$ is the unit vector normal to the sensing coil and $S_{S}$ is the sensing coil’s area.

### 2.2. Mutual Inductance Model

An alternative measurement model may be obtained by assuming the coils to be inclined circular filaments, arbitrarily positioned in space with respect to each other and by computing their mutual inductance *M* [16]. Let us consider the geometric configuration of the coils depicted in Figure 1. The sensing coil is assumed to be at the origin of the coordinate system, lying on the xy plane. The field-generating coil is placed at an arbitrary position $\left(x_{u},y_{u},z_{u}\right)$, lying on the plane λ≡ax+by+cz+d=0, with $\sqrt{a^{2}+b^{2}+c^{2}}=1$. Therefore, as in Section 2.1, the vector $n_{G}={\left[a,b,c\right]}^{T}$ is the unit normal vector of the plane λ and describes the orientation of the field-generating coil. This vector is aligned with the coil’s axis. The mutual inductance between the field-generating coil and the sensing coil is given by [16]:

(4) $$M\left(x_{u},y_{u},z_{u},a,b,c\right)=\xi \int_{0}^{2\pi }\frac{\left[p_{1}\cos\varphi +p_{2}\sin\varphi +p_{3}\right]\Psi \left(k\right)}{k\sqrt{V_{0}^{3}}}\;d\varphi$$

where $\xi =\frac{\mu_{0}R_{s}}{\pi }$, $p_{1}=\pm \frac{\gamma c}{\ell }$, $p_{2}=\mp \frac{\beta \ell^{2}+\gamma ab}{\ell L}$, $p_{3}=\frac{\alpha c}{L}$, $\ell =\sqrt{a^{2}+b^{2}}$, $L=\sqrt{a^{2}+b^{2}+c^{2}}$, $k=\sqrt{\frac{4V_{0}}{A_{0}+2V_{0}}}$,

$$\begin{array}{cc} V_{0}^{2}= & \alpha^{2}\left[\left(1-\frac{b^{2}c^{2}}{\ell^{2}L^{2}}\right)\cos^{2}\varphi +\frac{c^{2}}{\ell^{2}}\sin^{2}\varphi +\frac{abc}{\ell^{2}L}\sin2\varphi \right]\\ & +\beta^{2}+\gamma^{2}\mp 2\alpha \frac{\beta ab-\gamma \ell^{2}}{\ell L}\cos\varphi \mp \frac{2\alpha \beta c}{\ell }\sin\varphi , \end{array}$$

$$\begin{array}{c} A_{0}=1+\alpha^{2}+\beta^{2}+\gamma^{2}+\delta^{2}+2\alpha \left(p_{4}\cos\varphi +p_{5}\sin\varphi \right), \end{array}$$

$$\begin{array}{c} \alpha =\frac{R_{G}}{R_{S}},\beta =\frac{x_{u}}{R_{S}},\gamma =\frac{y_{u}}{R_{S}},\delta =\frac{z_{u}}{R_{S}}, \end{array}$$

where $R_{S}$ is the radius of the sensing coil, assumed to be in the origin, and $R_{G}$ is the radius of the field-generating coil. The choice of the sign in the above equations must be made consistently with the displayed convention, i.e., either all upper signs or all lower signs should be used. In Label (4), $\Psi \left(k\right)=\left(1-\frac{k^{2}}{2}\right)K\left(k\right)-E\left(k\right)$, where K(k) is the complete elliptic function of the first kind and E(k) is the complete elliptic function of the second kind [16].

The computation of the elliptic integrals is typically performed using burdensome numerical methods, thus reducing the applicability of this measurement model to practical real-time tracking scenarios. To mitigate this problem, numerous approximation methods have been proposed to compute the elliptical integrals [20]. In this paper, we use the following polynomial approximations [21]:

$$\begin{array}{c} K\left(k\right)=\left[a_{0}+a_{1}k_{1}+\cdots +a_{4}k_{1}^{4}\right]-\left[b_{0}+b_{1}k_{1}+\cdots +b_{4}k_{1}^{4}\right]\ln\left(\frac{1}{k_{1}}\right)+\epsilon_{K}\left(k\right),\;0\leq k<1, \end{array}$$

where $k_{1}=1-k$, the approximation error term obeys $\left|\epsilon_{K}\left(k\right)\right|<2\times 10^{-8}$, and the coefficients of the polynomials are

$$\begin{array}{ccccccccc} a_{0}=1.38629436112, & & a_{1}=0.09666344259, & & a_{2}=0.03590092383, & & a_{3}=0.03742563713, & & a_{4}=0.01451196212\\ b_{0}=0.5, & & b_{1}=0.12498593597, & & b_{2}=0.06880248576, & & b_{3}=0.03328355346 & & b_{4}=0.00441787012, \end{array}$$

$$\begin{array}{c} E\left(k\right)=\left[1+c_{1}k_{1}+\cdots +c_{4}k_{1}^{4}\right]-\left[d_{1}k_{1}+\cdots +d_{4}k_{1}^{4}\right]\ln\left(\frac{1}{k_{1}}\right)+\epsilon_{E}\left(k\right), \end{array}$$

where the coefficients of the polynomials are

$$\begin{array}{ccccccc} c_{1}=0.44325141463, & & c_{2}=0.06260601220, & & c_{3}=0.03590092383, & & c_{4}=0.01736506451,\\ d_{1}=0.24998368310, & & d_{2}=0.09200180037, & & d_{3}=0.04069697526, & & d_{4}=0.00526449639, \end{array}$$

and the approximation error term obeys $\left|\epsilon_{E}\left(k\right)\right|<2\times 10^{-8}$.

The mutual inductance binds the rms voltage on the field-generating coil $V_{G}$ and that measured on the sensing coil $V_{S}$ through a constant, denoted by *C*, as follows [22]:

(5) $$\begin{array}{c} V_{S}\left(x_{u},y_{u},z_{u},a,b,c\right)=CV_{G}M\left(x_{u},y_{u},z_{u},a,b,c\right), \end{array}$$

The constant *C* can be estimated using a calibration procedure, described in the following section. Equation (5) represents the measurement model based on mutual inductance.

## 3. Numerical Simulation of a Magnetic Positioning System

To compare the positioning accuracy performance obtained by using the magnetic dipole model to that obtained by using the mutual inductance model, numerical simulations were performed. Noisy measurement data were created using different values of the signal-to-noise ratio, in order to characterize the influence of measurement noise on the accuracy performance.

### 3.1. Simulation Data

The simulation was performed by considering a grid of $N_{S}$ sensing coils lying on the xy plane and having fixed and known positions and orientation. Then, a set of positions and orientations of the field-generating coil, i.e., the true trajectory, was generated. For each element of this set, accurate values of the mutual inductance between the field-generating coil and the sensing coils were computed according to the model in [17,18]. Such model is more sophisticated than that in [16] described in Section 2.2 of this paper, since it takes into account all parameters related to the physical dimensions of the coils. According to this model, the finite dimension of the coils is taken into account by discretizing them into a mesh of filaments. This procedure is also known in the literature as the filament method [23]. Then, the total magnetic flux linked with an arbitrary filament at the sensor coil is calculated as the sum of the magnetic fluxes due to all the filaments in the field-generating coil. This model is characterized by a good accuracy since it can correctly account for the physical dimensions of the coils. However, this comes at the cost of a high computational complexity, due to the large number of required computations of the flux between every combination of primary and secondary filaments. Thus, it is unsuitable for real-time position estimation. Therefore, we used this model only to generate realistic simulation data. Instead, for the actual position estimation, we applied the simplified mutual inductance model described in Section 2.2, which approximates the entire coils as filaments thus reducing computational complexity.

The voltage at the sensing coil was simulated using an equivalent circuit similar to the one shown in [22]. Specifically, this circuital configuration exploits the resonance of an inductor-capacitor (LC) circuit, comprised of a capacitor connected in parallel with the coil. In the simulations, a resonant frequency of approximately $f_{0}=200$ kHz was considered. This resonant circuit is employed both at the field-generating coil and at the sensing coil, resulting in a sensed voltage that is greater than that obtained without the resonant circuit. Finally, white Gaussian noise having a standard deviation of $\sigma_{AWGN}$ was added to the simulated samples of the voltage signal received at each sensing coil. A record of $N=10^{3}$ samples of the noisy signal was generated for each sensing coil, with a sampling frequency of 4 MSa/s, and the root-mean-square value of this record was calculated, thus obtaining a simulation of the noisy voltage induced at each coil. The simulation was repeated for the entire trajectory using different values of $\sigma_{AWGN}$, in order to evaluate the influence of signal-to-noise ratio (SNR) on performance.

Once the noisy voltage data were simulated, they were used to estimate the vector parameter $\theta ={\left[x_{G},y_{G},z_{G},a,b,c\right]}^{T}$, which represents the position and orientation of the field-generating coil. The estimate of the vector parameter θ, which we denote as $\hat{\theta }={\left[{\hat{x}}_{G},{\hat{y}}_{G},{\hat{z}}_{G},\hat{a},\hat{b},\hat{c}\right]}^{T}$, was obtained by fitting the noisy voltage data simulated at the sensing coils to the corresponding voltages predicted by the two models in Section 2.1 and Section 2.2 using a nonlinear least squares approach as follows:

(6) $$\begin{array}{c} \hat{\theta }=\underset{\theta }{arg min}\sum_{i=1}^{N_{S}}{\left({\tilde{V}}_{rms,i}-V_{S,i}\left(\theta \right)\right)}^{2}, \end{array}$$

where ${\tilde{V}}_{rms,i}$ is the rms voltage measured at the sensing coil *i* and $V_{S,i}\left(\theta \right)$ is the predicted voltage at sensing coil *i*, obtained by simulation and corrupted by noise, and $V_{S,i}\left(\theta \right)$ is the predicted voltage at sensing coil *i*, obtained alternatively using the model in Label (3) or that in Label (5). The optimization method used was the Nelder–Mead simplex method [24].

**Figure 2:**
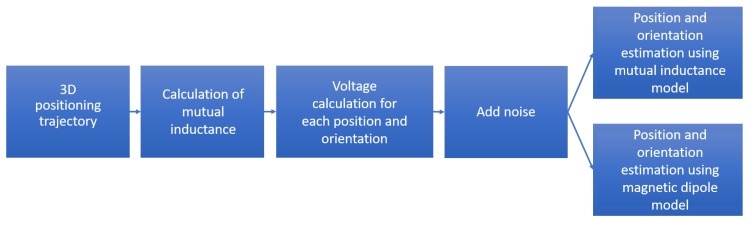
Numerical simulation procedure.

At each trajectory point, the initial guess of the optimization routine was provided by the position and orientation estimated at the previous trajectory point. At the first trajectory point, such initial guess was provided by the true values of the position and orientation, to emulate the situation in which the trajectory of the field generating coil starts from a known position and orientation. A block diagram illustrating the simulation procedure is shown in Figure 2.

### 3.2. Calibration

The methods described in Section 2.1 and Section 2.2 require a preliminary calibration phase to calculate the scale factor that establishes the relationship between the magnetic field or mutual inductance and measured voltage. For the mutual inductance model, such scale factor is denoted as *C* in (5). On the other hand, for the magnetic dipole model, such scale factor is theoretically known from Equation (3). However, due to imperfections in the coil construction, parasitic effects, and other nonideality factors, in a practical scenario it deviates from its theoretical value. Thus, the calibration procedure is performed in order to determine it empirically. In order to be consistent between experiments and simulations, we perform this procedure in both simulations and experiments.

**Figure 3:**
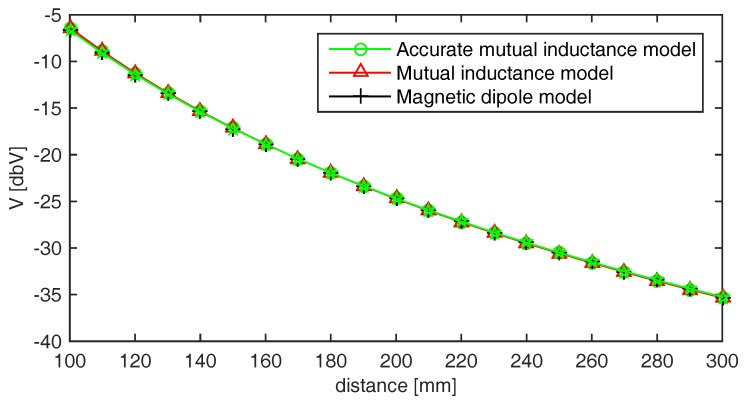
Preliminary calibration performed before the numerical simulation.

The calibration procedure, as in [8], consists of taking voltage measurements at known positions and orientations between the coils. This procedure was performed, in the simulations as well as in the experiments, by placing the field-generating coil and the sensing coil at a set of known distances, with both coils lying on the same plane. At each distance, one measurement result was taken and used to calculate the ratio between the measured voltage and the modeled voltage. Then, the average of the ratios at all distances was used as the calibration constant. The results obtained in simulation are shown in Figure 3, demonstrating a good agreement between the considered models after the calibration procedure.

### 3.3. Simulation Setup and Performance Evaluation Metrics

**Figure 4:**
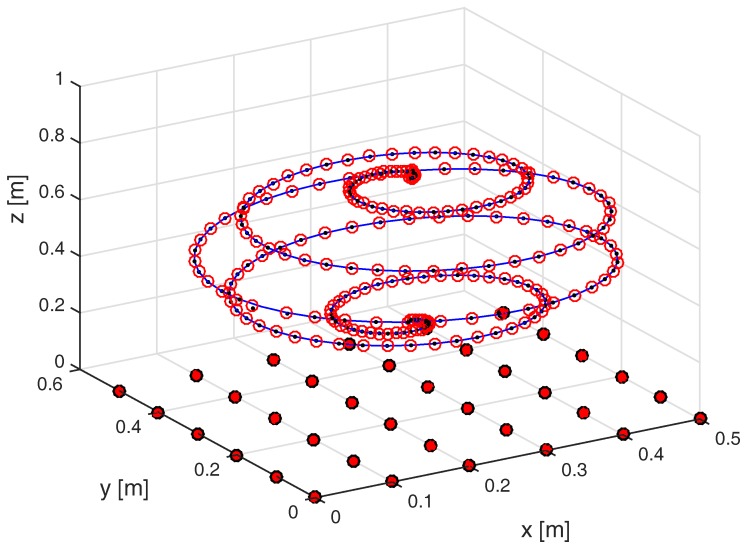
Trajectory used for the numerical simulations. Sensing coils are placed on the xy plane and denoted by red-filled circlets. The true trajectory of the field-generating coil is marked as a solid blue line, the trajectory estimated by the mutual inductance method by black dots, and that estimated by the magnetic dipole method by red circlets.

In the case of the numerical simulation, the 200-point trajectory in the 3D space shown in Figure 4 was used. Furthermore, a 2D-array comprised of 36 sensing coils was employed. Although, in the general case, the position of the sensor coils could be decided arbitrarily, a planar 2D array with uniform placement of the coils provides advantages in terms of simplicity of the construction of the experimental setup. Seven values of the standard deviation of the Gaussian noise $\sigma_{AWGN}$ were used, resulting in a set of mean SNR values (averaged over the entire trajectory) ranging from 13 dB to 100 dB.

The positioning error *d* was defined as the Euclidean distance between the true position and the estimated position as follows:

(7) $$d=\sqrt{{\left(x_{G}-{\hat{x}}_{G}\right)}^{2}+{\left(y_{G}-{\hat{y}}_{G}\right)}^{2}+{\left(z_{G}-{\hat{z}}_{G}\right)}^{2}}.$$

Moreover, the orientation error ϕ was defined as the angle between the true unit normal vector of the plane where the field generating coil lies, i.e., $n_{G}=\left[a\;b\;c\right]$, and the corresponding estimate ${\hat{n}}_{G}=\left[\hat{a}\;\hat{b}\;\hat{c}\right]$, as follows:

(8) $$\phi =\arccos\left(\frac{n_{G}\cdot {\hat{n}}_{G}}{∥n_{G}∥∥{\hat{n}}_{G}∥}\right).$$

### 3.4. Numerical Simulation Results

In order to identify the most effective positioning strategy, the mutual inductance model and the magnetic dipole model were compared, assuming a slowly moving field-generating coil, such that the distance between the position at time *k* and that at time k+1 is on the order of millimeters. If such assumption is satisfied, we can approximate the amplitude of the sinusoid acquired at each record as constant.

**Figure 5:**
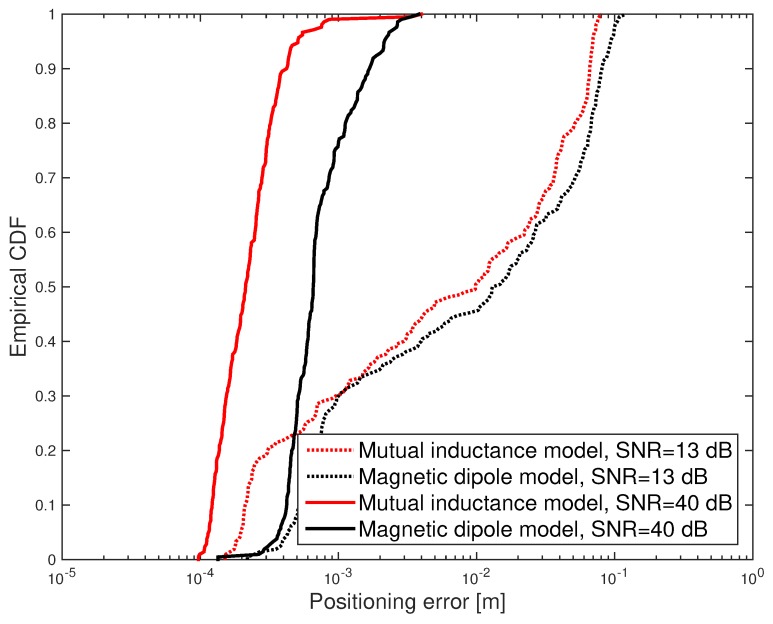
Simulation results: CDF of the positioning error obtained for SNR = 13 dB and SNR = 40 dB.

**Figure 6:**
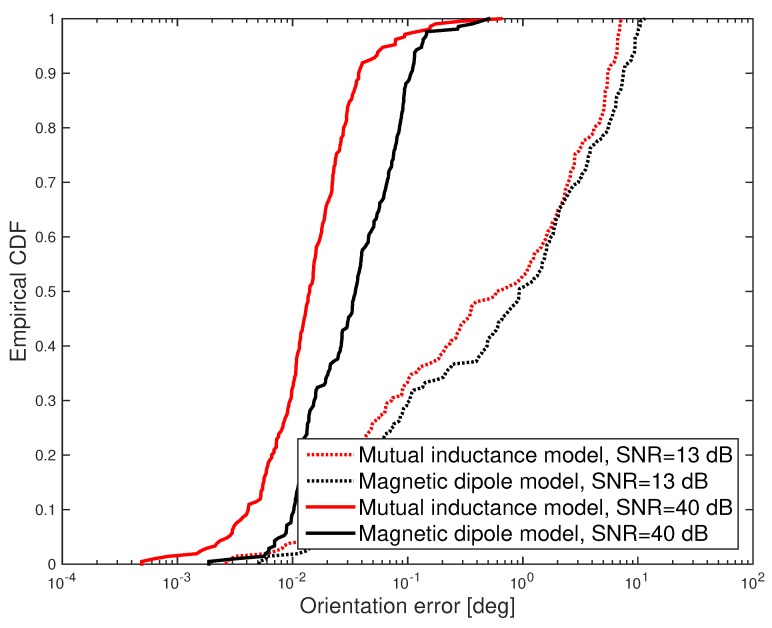
Simulation results: CDF of the angular error obtained for SNR = 13 dB and SNR = 40 dB.

**Figure 7:**
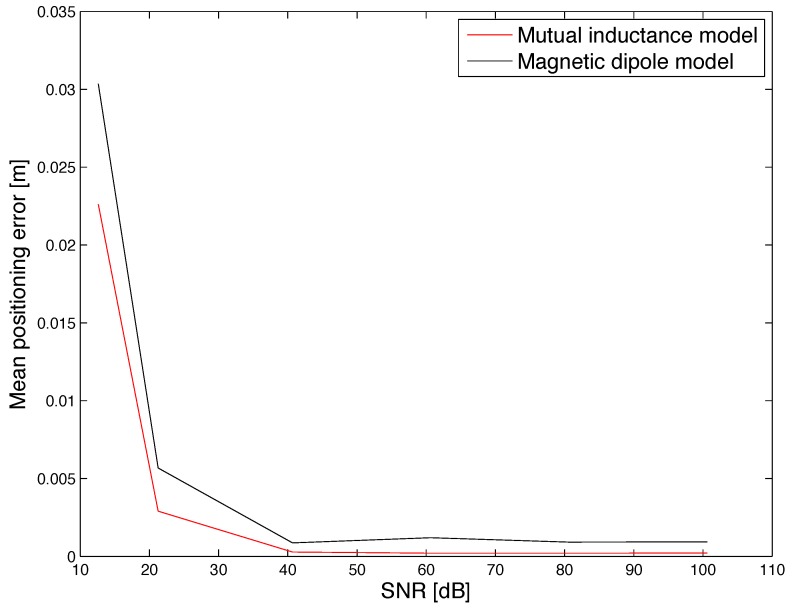
Simulation results: comparison of the mean positioning error obtained for different values of the SNR.

**Figure 8:**
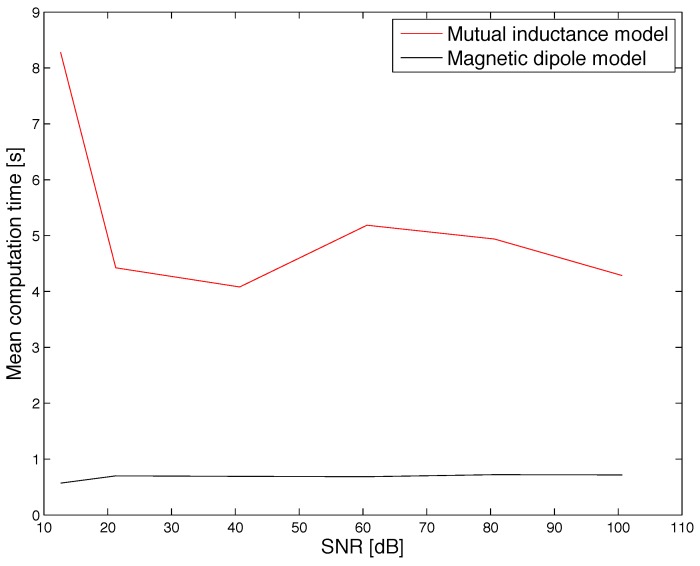
Simulation results: comparison of the mean computation time obtained for different values of the SNR.

Under such conditions, the cumulative distribution function (CDF) of the positioning error was evaluated and shown in Figure 5 and Figure 6. Furthermore, the mean positioning error obtained for different values of the SNR is shown in Figure 7. Results show that the mutual inductance model provides higher accuracy than the magnetic dipole model in terms of positioning and orientation error. Nevertheless, from the mean computation time plotted in Figure 8 it can be noticed that the magnetic dipole model has lower computational complexity than the mutual inductance model. As an example, for SNR = 81 dB, the computation time of the magnetic dipole model is approximately seven times smaller than that of the mutual inductance model as shown in Table 1. These computation times were measured on Matlab (Mathworks, Natick, MA, USA) implementations running on the same computer, a MacBook Pro (Apple, Cupertino, California, USA) with a Core i7 2.5 GHz processor and 16 Gb of RAM.

As shown in Table 1, the magnetic dipole moment has a computational time that is smaller than the computation time of the mutual inductance model by a factor 7. Furthermore, the accuracy degradation obtained when using the dipole model (a factor 4) is acceptable in practical situations, given that the error in simulation is below 1 mm. This trade-off between accuracy and computational time was used to guide the design process of the experiemntal prototype. Specifically, given its strong advantage in terms of computation time, the magnetic dipole moment was selected for implementation in the prototype, as described in the following section.

## 4. Experimental Magnetic Tracking System (MTS)

By analyzing the results of the numerical comparison provided in the previous section, we observed that the magnetic dipole model provides a better trade-off between accuracy and computational complexity. For this reason, it may be more suited for realizing practical positioning and tracking systems. Based on this observation, we chose to employ the magnetic dipole model for realizing a complete experimental tracking system. Such a system requires the solution of nonlinear problems, which in general may result in high computational complexity. However, with the rapid development of the microprocessor, it is possible to perform computational tasks of increasing complexity, therefore algorithms for solving nonlinear problems have become more appealing. For nonlinear state-space models, it is not possible in general to derive the optimal state estimator in closed form, but various modifications of the Kalman filter were proposed to estimate the state. The Unscented transformation method, presented in [25,26], may be used to propagate mean and covariance information through nonlinear transformations. The Unscented transformation is the base of the Unscented Kalman Filter (UKF), which was used for the experimental prototype realization described in the remainder of this paper.

### 4.1. Hardware Setup

**Figure 9:**
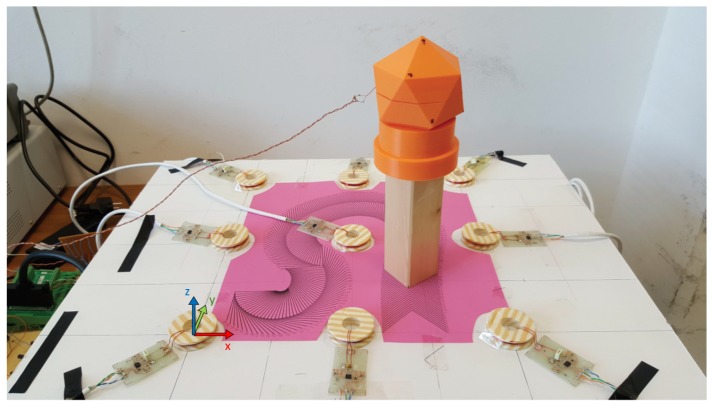
Hardware setup: nine sensing coils are placed on the xy plane shown at the bottom of the picture. Each of them is connected to a printed circuit board containing a parallel capacitor and an instrumentation amplifier. The field-generating coil is placed inside the icosahedral support shown at the top of the picture. The origin of the coordinate system is in the center of the coil shown at the bottom left of the picture, where a diagram of the axes of the coordinate system is also depicted.

A prototype positioning system was designed and implemented, using the magnetic dipole model. Nine sensing coils were realized using enameled copper wire and placed on the same plane as shown in Figure 9, so that they had fixed and known positions and orientations. The radius of the sensing coils is 18.5 mm, the number of turns is 5, the coils’ thickness is 0.5 mm and the coils’ height is 3.5 mm. The field-generating coil has a radius of 5 mm, the number of turns is 15, the thickness is 0.5 mm and height is 3.5 mm. The number of turns of the coils was selected such that it resulted in a sufficiently high amplitude of the sensed voltage. Other choices are possible, such as a larger number of turns. However, this choice would increase the physical dimension of the coils and the construction complexity. A larger number of turns would also increase resistance, thus deteriorating the quality factor of the resonators. The resulting nominal inductance of the coils is $L_{1}=L_{2}=L=2\;\mu$H. Similarly to the configuration simulated in Section 3, to reduce current consumption at the transmitter side and increase the operating range, each coil was employed in a resonator configuration, by connecting it in parallel with a capacitor having the same nominal value of $C_{1}=C_{2}=C=330$ nF. The resonant frequency of the realized circuit was approximately 184 kHz.

The voltage induced at each sensing coil was amplified by an AD8421 instrumentation amplifier. The output of each amplifier was connected to one of the channels of an U2331A data acquisition board, where the signals were digitized at 3 MSa/s, 12 bits. The duration of the observation window used to obtain the digitized record of the signal was 100 μs. Digitized signals were transferred to a host PC for further processing.

In order to realize known orientations of the field-generating coil, it was placed inside a 3D-printed support shaped as an icosahedron. By changing the orientation of such a support, it is possible to place the field-generating coil in a set of predefined orientations. Furthermore, the icosahedral support was placed on a wooden stand that was then aligned to a set of printed reference markings. Such markings define a 199-point reference trajectory where the distance between consecutive points is 3 mm. The combined use of the printed reference markings and the icosahedral support allowed obtaining ground truth information for both position and orientation.

Notice that the sensing coils span a region shaped as a square having a 30-cm side. Having verified that the system can detect the mobile node’s signal up to a height of 30 cm, this results in an operational volume of approximately 30×30×30 cm^3^. In order to cover a larger operational volume, it would be necessary either to use a larger number of sensors or to realize field-generating coils having larger physical dimensions.

The experimental tests were conducted by placing the field-generating coil at each point of the reference trajectory and acquiring a data record from all sensing coils. The test was repeated for two different positions of the icosahedral support, corresponding to two different initial orientations of the field-generating coil. The first initial orientation was defined by coefficients a=0, b=0 and c=1, thus the field-generating coil was parallel to the sensing coils. The second orientation was defined by coefficients a=0.72361, b=−0.52573 and c=0.44721, thus the field-generating coil was inclined with respect to the sensing coils. Note that, during the second test, the orientation of the field-generating coil changed at each trajectory point.

Prior to the experimental tests, the calibration procedure was performed. As in [8], it consisted of taking voltage measurements at known positions and orientations between the coils for calculating the calibration constants. It was performed experimentally by placing the field-generating coil and each of the sensing coils at a known distance of 150 mm in a coaxial configuration.

During the experimental tests, tracking was performed using the algorithm described in the following section.

### 4.2. Tracking Algorithm

To perform tracking, we define a state-space dynamic model, where the state vector x is given by the position, velocity, and orientation of the field-generating coil, as follows:

(9) $$x={\left[x_{G}\;y_{G}\;z_{G}\;v_{x}\;v_{y}\;v_{z}\;a\;b\;c\right]}^{T}$$

with $v_{x}$, $v_{y}$, and $v_{z}$ denoting the components of the velocity vector associated with the field-generating coil in the 3D space.

The tracking model adopted is a nearly constant velocity model [27] with the following state-space equations

(10) $$\begin{array}{c} \begin{array}{cc} x_{k+1} & =Fx_{k}+w_{k},\\ z_{k} & =h\left(x_{k}\right)+e_{k}, \end{array} \end{array}$$

where *k* is the time step, w is white Gaussian process noise with covariance matrix *Q*, e is white Gaussian measurement noise with covariance matrix *R*, z is the measured voltage at sensing nodes, and h is the nonlinear measurement function that relates the state vector to the measured voltages, corresponding to the magnetic dipole measurement model given by Label (3).

The state-transition matrix is given by:

$$F=\left[\begin{array}{ccccccccc} 1 & 0 & 0 & T & 0 & 0 & 0 & 0 & 0\\ 0 & 1 & 0 & 0 & T & 0 & 0 & 0 & 0\\ 0 & 0 & 1 & 0 & 0 & T & 0 & 0 & 0\\ 0 & 0 & 0 & 1 & 0 & 0 & 0 & 0 & 0\\ 0 & 0 & 0 & 0 & 1 & 0 & 0 & 0 & 0\\ 0 & 0 & 0 & 0 & 0 & 1 & 0 & 0 & 0\\ 0 & 0 & 0 & 0 & 0 & 0 & 1 & 0 & 0\\ 0 & 0 & 0 & 0 & 0 & 0 & 0 & 1 & 0\\ 0 & 0 & 0 & 0 & 0 & 0 & 0 & 0 & 1 \end{array}\right],$$

where *T* is the time interval between two consecutive time steps.

The process noise covariance matrix is

$$Q={\left[\begin{array}{ccccccccc} 10^{-6} & 0 & 0 & 0 & 0 & 0 & 0 & 0 & 0\\ 0 & 10^{-6} & 0 & 0 & 0 & 0 & 0 & 0 & 0\\ 0 & 0 & 10^{-6} & 0 & 0 & 0 & 0 & 0 & 0\\ 0 & 0 & 0 & 10^{-3} & 0 & 0 & 0 & 0 & 0\\ 0 & 0 & 0 & 0 & 10^{-3} & 0 & 0 & 0 & 0\\ 0 & 0 & 0 & 0 & 0 & 10^{-3} & 0 & 0 & 0\\ 0 & 0 & 0 & 0 & 0 & 0 & 20 & 0 & 0\\ 0 & 0 & 0 & 0 & 0 & 0 & 0 & 20 & 0\\ 0 & 0 & 0 & 0 & 0 & 0 & 0 & 0 & 20 \end{array}\right]}^{2}.$$

Furthermore, the measurement noise covariance matrix is given by

$$R=\sigma_{n}^{2}I_{9},$$

where $\sigma_{n}=2.5\times 10^{-3}$, and $I_{9}$ denotes the 9×9 identity matrix. The value of the standard deviation of the measurement noise, i.e., $\sigma_{n}$, was defined by processing the experimental data acquired using the hardware setup.

The UKF algorithm is an extension of the Kalman filter that reduces the linearization errors of the extended Kalman filter (EKF). Unlike the Kalman filter and EKF, which propagate state estimates, the UKF propagates an estimate of the probability density function of the state and the measurement through the nonlinear function. With respect to the EKF, the UKF may be applied to models that are not easily differentiable.

The UKF deterministically extracts so-called sigma points from the Gaussian distribution and passes these through a nonlinear function, in our case the measurement function $h(x_{k})$ in Label (10). For an *n*-dimensional Gaussian distribution with mean μ and covariance Σ the sigma points χ, which are *n*-dimensional vectors, are chosen according to following rule [26,28]:

$$\begin{array}{lll} \chi_{0}= & \mu ,\\ \chi_{i}= & \mu +{\left(\sqrt{\left(n+\lambda \right)\Sigma }\right)}_{i} & \mathrm{for}\;i=1,\ldots ,n,\\ \chi_{i}= & \mu -{\left(\sqrt{\left(n+\lambda \right)\Sigma }\right)}_{i-n} & \mathrm{for}\;i=n+1,\ldots ,2n, \end{array}$$

and the corresponding weights *w* are defined as follows:

$$\begin{array}{lll} cllw_{m}^{\left[0\right]}= & \lambda /(n+\lambda ),\\ w_{c}^{\left[0\right]}= & \lambda /\left(n+\lambda \right)+\left(1-\xi^{2}+\rho \right),\\ w_{m}^{\left[i\right]}= & w_{c}^{\left[i\right]}=1/\left[2\left(n+\lambda \right)\right] & \mathrm{for}\;i=1,\ldots ,2n, \end{array}$$

where $\lambda =\xi^{2}\left(n+\kappa \right)-n$ is a factor, expressed as a function of the scaling parameters ξ and κ, that determines how the sigma points are spread from the mean of a Gaussian distribution, and ρ is an additional parameter used to incorporate prior knowledge on the distribution.

These sigma vectors are propagated through the nonlinear function

$$\Upsilon^{\left[i\right]}=h\left(\chi^{\left[i\right]}\right),\;i=1,\ldots ,2n$$

The mean and covariance for z, denoted as $\hat{z}$ and $P_{z}$, respectively, are approximated using a weighted sample mean and covariance of the posterior sigma points:

$$\hat{z}\approx \sum_{i=0}^{2n}w_{m}^{\left[i\right]}\Upsilon_{i}$$

$$\begin{array}{c} P_{z}\approx \sum_{i=0}^{2n}w_{c}^{i}\left\{\Upsilon^{\left[i\right]}-\hat{z}\right\}{\left\{\Upsilon^{\left[i\right]}-\hat{z}\right\}}^{T}. \end{array}$$

Then, at each time step, the estimate of the state and the corresponding covariance matrix are obtained using recursive equations based on those of the Kalman filter. An extensive explanation of the UKF algorithm is given by [26,28].

During the experimental tests, at the first time step, the state estimate was initialized at the true value of the state. At subsequent time steps, the state was estimated recursively using the UKF algorithm, to produce an estimated trajectory.

### 4.3. Experimental Results and Discussion

**Figure 10:**
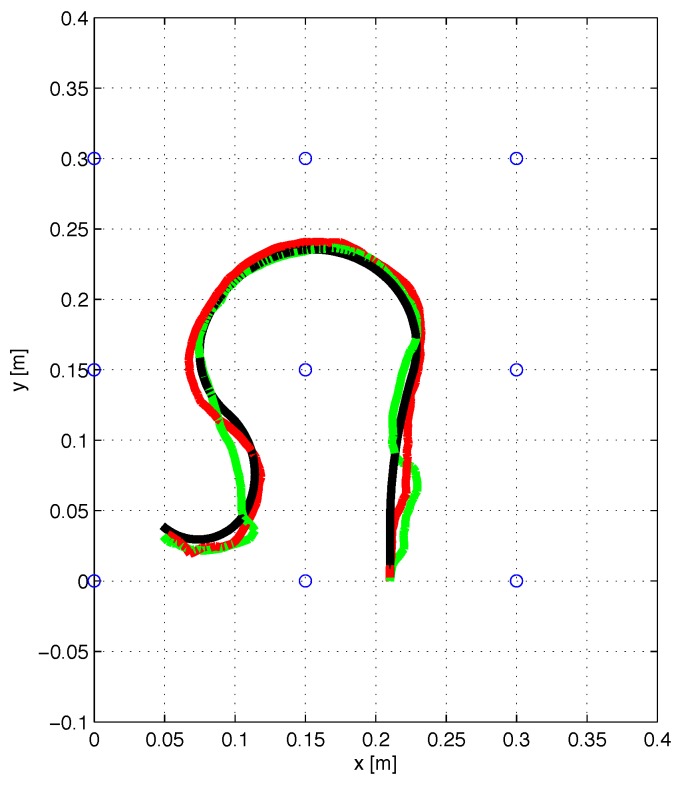
Experimental results: position estimation under two orientation configurations. The black line is the true trajectory, the green line is the estimated trajectory with the first configuration, i.e., initial coil orientation a=0, b=0 and c=1, the red line is the estimated trajectory with the second configuration, i.e., coil orientation a=0.72361, b=−0.52573 and c=0.44721.

An overhead view of the true 3D trajectory that was used for experimental tests is shown in Figure 10, together with the estimated trajectories for the two orientation configurations tested. The coordinates of the initial point are [0.21000.203], on the bottom right part of Figure 10. Visually, a good qualitative agreement between the true trajectory and the estimated one may be observed.

**Figure 11:**
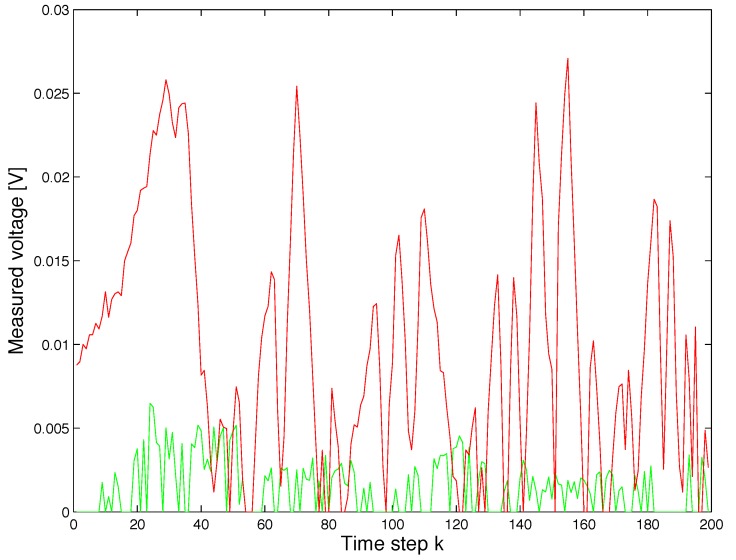
Plot illustrating the minimum voltage that was experimentally measured along the path in a set of 199 positions inside the test area. The green line is obtained using the initial orientation of transmitter coil a=0, b=0 and c=1 and the red line using the initial orientation a=0.72361, b=−0.52573 and c=0.44721.

**Figure 12:**
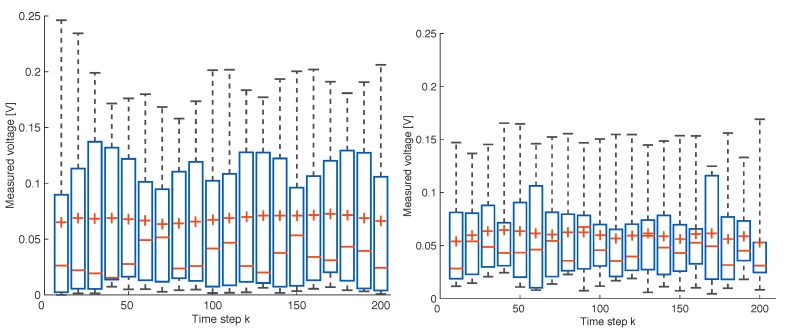
Boxplot illustrating the experimental results of measurements along the paths in a set of 20 positions inside the test area. The results of the test performed with the initial orientation of transmitter coil a=0, b=0 and c=1 are shown in the figure on the left and those with the initial orientation a=0.72361, b=−0.52573 and c=0.44721 in the figure on the right. In each box, the central mark is the median, the box edges denote the 25th and 75th percentiles, and the top and bottom ends of the dashed lines are the maximum and minimum values, respectively. The cross denotes the mean.

We can notice that the voltage measured is strongly dependent on the position and orientation of the coil. As an example, in the first configuration, the minimum voltage measured is always lower than that measured in the second configuration, as shown in Figure 11. Furthermore the variance of the voltage measured in the first configuration is larger than the second one, as shown in Figure 12. We recall here that, as described in Section 4.1, in the second orientation configuration, the field generating coil’s orientation changes with respect to the absolute reference at each trajectory point. Instead, in the first orientation configuration, the orientation does not change and the coil always remains parallel to the xy plane.

**Figure 13:**
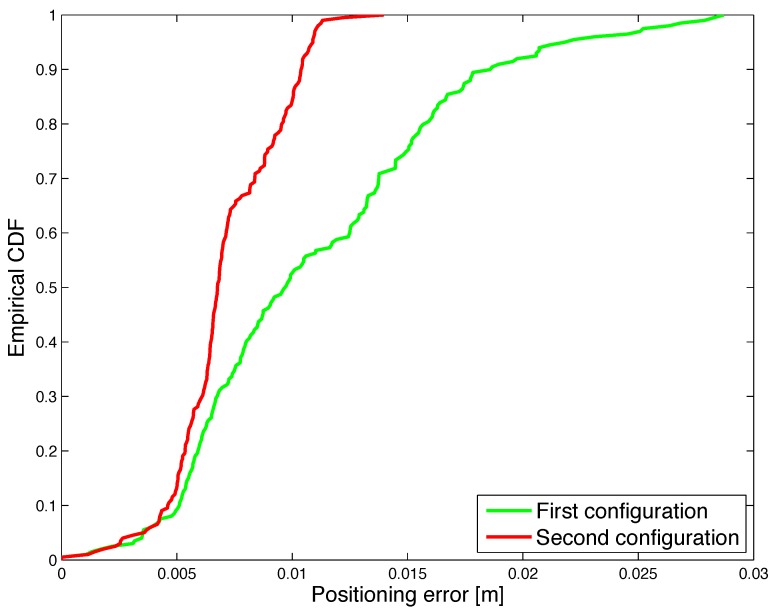
Experimental results: CDF of the positioning error under two orientation configurations. The green line (first configuration) is related to the test performed with initial orientation of transmitter coil a=0, b=0 and c=1, the red line (second configuration) is related to the test performed with the initial orientation of transmitter coil a=0.72361, b=−0.52573 and c=0.44721.

**Figure 14:**
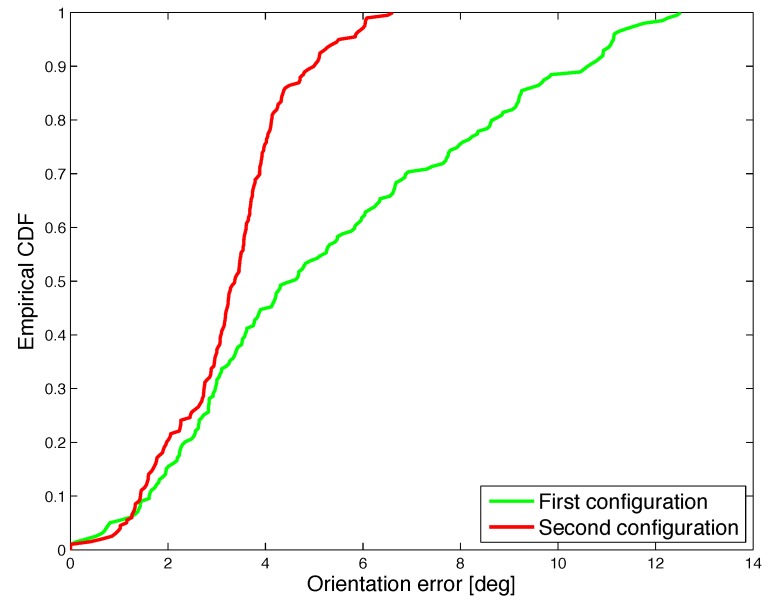
Experimental results: CDF of the orientation error under two orientation configurations. The green line (first configuration) is related to the test performed with the initial orientation of transmitter coil a=0, b=0 and c=1, the red line (second configuration) is related to the test performed with the initial orientation of transmitter coil a=0.72361, b=−0.52573 and c=0.44721.

In order to quantitatively evaluate and compare the performance under different orientation configurations, the CDF of the positioning error and orientation error are shown in Figure 13 and Figure 14.

Results show that the performance remains satisfactory for different coil orientation configurations in 3D space, therefore proving the robustness of the realized system. The median positioning error is 9.7 mm for the first orientation configuration, i.e., parallel coils, and 6.8 mm for the second orientation configuration. Furthermore, for the first orientation configuration, in 90% of the cases the positioning error is less than 10 mm, while the orientation error is smaller than 5 degrees. For the second orientation configuration, the estimated position shows an error of less than 20 mm in 90% of the cases and the orientation error is less than 11 degrees.

The average computation time of an iteration of the UKF tracking algorithm is approximately 26 ms, therefore proving the feasibility of real-time tracking.

By comparing the experimental results with those of the numerical simulations in Section 3, it is possible to notice that the positioning error is larger in the experimental results by a factor of approximately 10, with the simulation results achieving an error below 1 mm. This discrepancy is due mainly to nonidealities present in the experimental setup. The main aspect influencing the setup nonideality is the uncertainty of the ground truth information provided by the experimental setup. Such setup is in fact affected by an error that may be quantified to be of a few millimeters, due to the difficulty in physically realizing structures and supports capable of placing the coils in predetermined positions with submillimeter accuracy.

The results obtained may be compared to previous work on magnetic tracking systems by taking several aspects into account, including operational range, accuracy, and deployment complexity. Specifically, in [9], an average error of 3.3 mm was observed when measuring the distance between a triaxial transmitting coil and an uniaxial receiving coil, which was not stationary but was mounted on a rotating device. Compared to [9], our approach is operational also in stationary conditions and provides estimates of 3D positioning and orientation. Moreover, in [10], a tracking system with three triaxial generating coils and two triaxial sensor coils was described. This system achieved a maximum positioning error below 3 mm and an average positioning error of approximately 1 mm. Compared to this system, the setup we considered in this paper allows for a lower-complexity implementation and deployment by eliminating the requirement for realizing triaxial coils, which are prone to construction imperfections and axis misalignment, at the expense of a worse accuracy. In [11] a system for tracking body movements was realized using three orthogonal generating coils, magnetic-field sensors, and inertial sensors. The system was designed to be portable and fixed to the body. The observed tracking accuracy was approximately 5 mm. Compared to this system, the infrastructure of our realized prototype provides similar accuracy but has a different purpose, since it is not designed to be worn but rather to be placed in an environment where a mobile device can be tracked.

Furthermore, a comparison with other tracking technologies might be considered. In particular, the application of the Bluetooth low energy (BLE) technology for indoor positioning purposes was investigated in [2] This technology offers the advantage of an easy implementation, because BLE functionality is embedded in widespread commercial devices. Compared to our prototype magnetic tracking system, its longer operational range allows for suitable application to many indoor scenarios. However, the attinable accuracy is worse, with a positioning error in the order of a meter.

Another technology that is potentially suitable for realizing tracking systems is ultrasound propagation. As an example, in [4] the ultrasonic technology was employed to realize a system that integrates the information obtained by a set of local positioning systems with odometry for mobile robot localization. This system achieved decimeter-order positioning accuracy over an operating range of several meters. In [5], a sub-centimeter positioning system based on ultrasonic sensors was presented. Compared with the magnetic tracking system presented in this paper, it is more vulnerable to obstructions and its performance might be negatively affected by direction-dependent sensors. Finally, compared to optical tracking systems, the magnetic-field technology provides robustness to non-line-of-sight conditions.

The presented experimental results obtained by using the realized prototype confirm that magnetic tracking is a viable choice for such applications as hand tracking for robotic telemanipulation and for biomedical applications. Specifically, these applications may benefit from the good tradeoff between ease of implementation, low computational complexity, accuracy, and robustness provided by the magnetic dipole model.

## 5. Conclusions

In this paper, a comparison of the magnetic dipole model and the mutual inductance model in the context of 3D positioning and tracking was presented. Numerical comparison results show that the mutual inductance model allows for a better positioning accuracy than the magnetic dipole model, but at the expense of a higher computation time. Therefore, the magnetic dipole model might be a more suitable choice in practical applications with constrained computational resources. After considering the trade-off between accuracy and computational complexity resulting from the two models, the magnetic dipole model was selected for the development of a 3D positioning and tracking system using a quasi-stationary magnetic field generated by coils. The realized prototype is a 9-sensor system. Measurement results were obtained in two orientation configurations. The median positioning error was 9.7 mm for the first orientation configuration, which used parallel coils, and 6.8 mm for the second orientation configuration, where the orientation of the field-generating coil changed at each trajectory point. Such results show the feasibility of tracking the position and orientation of a mobile coil in real time with a median positioning error below 1 cm and a worst-case error of the order of 2 cm and 11 degrees, inside a spatial region of 30 × 30 × 30 cm^3^ operational volume.

## Figures and Tables

**Table 1:** Numerical simulation results: mean computation time and mean positioning error for each position calculation, with SNR = 81 dB.

Model	Mean Computation Time [s]	Mean Positioning Error [mm]
Magnetic Dipole	0.72	0.91
Mutual Inductance	4.94	0.21

## References

[1] De Angelis G., Moschitta A., Carbone P. (2016). Positioning Techniques in Indoor Environments Based on Stochastic Modeling of UWB Round-Trip-Time Measurements. IEEE Trans. Intell. Transp. Syst..

[2] Castillo-Cara M., Lovón-Melgarejo J., Bravo-Rocca G., Orozco-Barbosa L., García-Varea I. (2017). An Empirical Study of the Transmission Power Setting for Bluetooth-Based Indoor Localization Mechanisms. Sensors.

[3] Gharghan S.K., Nordin R., Ismail M. (2016). A Wireless Sensor Network with Soft Computing Localization Techniques for Track Cycling Applications. Sensors.

[4] Gualda D., Ureña J., García J.C., Lindo A. (2014). Locally-Referenced Ultrasonic–LPS for Localization and Navigation. Sensors.

[5] De Angelis A., Moschitta A., Carbone P., Calderini M., Neri S., Borgna R., Peppucci M. (2015). Design and Characterization of a Portable Ultrasonic Indoor 3D Positioning System. IEEE Trans. Instrum. Meas..

[6] Pasku V., De Angelis A., De Angelis G., Arumugam D.D., Marco D., Paolo C., Antonio M., Ricketts D.S. (2017). Magnetic Field Based Positioning Systems. IEEE Commun. Surv. Tutor..

[7] Kasmi Z., Norrdine A., Blankenbach J. (2015). Towards a Decentralized Magnetic Indoor Positioning System. Sensors.

[8] Pasku V., De Angelis A., De Angelis G., Moschitta A., Carbone P. (2017). Magnetic Field Analysis for 3D Positioning Applications. IEEE Trans. Instrum. Meas..

[9] Song S., Qiao W., Li B., Hu C., Ren H., Meng M.Q.H. (2014). An Efficient Magnetic Tracking Method Using Uniaxial Sensing Coil. IEEE Trans. Magn..

[10] Hu C., Song S., Wang X., Meng M.Q.H., Li B. (2012). A Novel Positioning and Orientation System Based on Three-Axis Magnetic Coils. IEEE Trans. Magn..

[11] Roetenberg D., Slycke P.J., Veltink P.H. (2007). Ambulatory Position and Orientation Tracking Fusing Magnetic and Inertial Sensing. IEEE Trans. Biomed. Eng..

[12] Sun Z., Foong S., Maréchal L., Teo T.H., Tan U.X., Shabbir A. Using heterogeneous sensory measurements in a compliant magnetic localization system for medical intervention. Proceedings of the 2015 IEEE International Conference on Advanced Intelligent Mechatronics (AIM).

[13] Plotkin A., Paperno E. (2003). 3D magnetic tracking of a single subminiature coil with a large 2-D array of uniaxial transmitters. IEEE Trans. Magn..

[14] Kofman J., Wu X., Luu T.J., Verma S. (2005). Teleoperation of a robot manipulator using a vision-based human-robot interface. IEEE Trans. Ind. Electron..

[15] Meli L., Salvietti G., Gioioso G., Malvezzi M., Prattichizzo D. Multi-contact bilateral telemanipulation using wearable haptics. Proceedings of the 2016 IEEE/RSJ International Conference on Intelligent Robots and Systems (IROS).

[16] Babic S., Sirois F., Akyel C., Girardi C. (2010). Mutual Inductance Calculation Between Circular Filaments Arbitrarily Positioned in Space: Alternative to Grover’s Formula. IEEE Trans. Magn..

[17] Babic S.I., Akyel C. (2008). Calculating Mutual Inductance Between Circular Coils With Inclined Axes in Air. IEEE Trans. Magn..

[18] Babic S.I., Martinez J., Akyel C., Babic B. (2014). Mutual inductance calculation between misalignment coils for wireless power transfer of energy. Prog. Electromagn. Res. M.

[19] Moschitta A., De Angelis A., Dionigi M., Carbone P. Analysis of simultaneous 3D positioning and attitude estimation of a planar coil using inductive coupling. Proceedings of the 2017 IEEE International Instrumentation and Measurement Technology Conference (I2MTC).

[20] Fukushima T. (2009). Fast computation of complete elliptic integrals and Jacobian elliptic functions. Celest. Mech. Dyn. Astron..

[21] Abramowitz M., Stegun I.A. (1964). Section 17.3. Complete Elliptical Integrals of the First and Second Kinds. Handbook of Mathematical Functions with Formulas, Graphs, and Mathematical Tables.

[22] Dionigi M., De Angelis G., Moschitta A., Mongiardo M., Carbone P. (2014). A Simple Ranging System Based on Mutually Coupled Resonating Circuits. IEEE Trans. Instrum. Meas..

[23] Kim K.-B., Levi E., Zabar Z., Birenbaum L. (1997). Mutual inductance of noncoaxial circular coils with constant current density. IEEE Trans. Magn..

[24] Nelder J.A., Mead R. (1965). A Simplex Method fot Function Minimization. Comput. J..

[25] Julier S., Uhlmann J. (1997). A new extension of the Kalman filter to nonlinear systems. Int. Symp. Aerospace/Defense Sens. Simul. Controls.

[26] Wan E.A., Van Der Merwe R. The Unscented Kalman Filter for Nonlinear Estimation. Proceedings of the IEEE 2000 Adaptive Systems for Signal Processing, Communications, and Control Symposium (Cat. No.00EX373).

[27] Bar-Shalom Y., Li X., Kirubarajan T. (2004). Estimation with Applications to Tracking and Navigation: Theory Algorithms and Software.

[28] Thrun S., Burgard W., Fox D. (2005). Probabilistic Robotics (Intelligent Robotics and Autonomous Agents).

